# Telemedicine in Iran: Chances and Challenges

**Published:** 2013-01

**Authors:** Zeinab Salehahmadi, Fatemeh Hajialiasghari

**Affiliations:** 1 Information Technology, Bushehr, University Instructor, Tehran University of Medical Science, Tehran, Iran;; 2 Deputy of Health, Tehran University of Medical Science, Tehran, Iran;; 3 Director of Information Technology Department, Academy of Russian Sciences, Tajikestan

**Keywords:** Telemedicine, Advantages, Drawbacks, Iran

## Abstract

**BACKGROUND:**

Technology is likely to transform the way care is delivered at home and in the community. Telemedicine, the child of IT and Medicine sciences is the use of telecommunication equipment and information technology to provide clinical care to individuals at distant sites and the transmission of medical and surgical information and images needed to provide that care. Undoubtedly, the advantages outweight its disadvantages, but just like any other innovations, it has some drawbacks. The present study outlines telemedicine strong and weak points. In this regard a survey has been done in Tehran University of Medical Sciences in Iran.

**METHODS:**

Between 14^th^ May and 14^th^ August 2012, 90 medical specialist men and 42 women from different hospitals of Tehran Medical Sciences University, Iran were enrolled by a simple ran- dom sampling method. They all completed a questionnaire to consider what are telemedicine chances and challenges.

**RESULTS:**

Making use of telemedicine was a profitable alternative in remote, rural/urban places especially in new project of “family physician” presented by Iran Ministry of Health. The results also highlighted that security considerations was an inevitable challenge of telemedicine, while shorter hospital stays and reduced warm ischemic time, and reduced morbidity and mortality rates were telemedicine merits. Despite most previous studies results, telemedicine has been recognised as a cost-effective alternative. Cultural, language distinctions as well as the level of literacy were barriers on deploying tele- medicine. There was no strong evidence showing that using telemedicine caused a decrease in tactile feedback.

**CONCLUSION:**

We need to fully understand and consider various outcomes and challenges of telemedicine before applying it.

## INTRODUCTION

Telemedicine, (’Tele’ extracted from the Greek word ’Telos’ means remote and ‘Medicine’ comes back to Latin word ‘mederi’ means ‘cure’) the use of telecommunication and information technologies in order to provide clinical health care at a distance, came into medical dictionary in 1920. Even if the term telemedicine has a more recent origin, but the concept is a relatively old one. It comes back to 90 years ago when Einthoven in 1906 published his work with distant consultation on electrocardiography. In Norway, the Haukeland hospital in the city of Bergen, pioneered in 1922 with medical consultations between ships and the hospital by using Bergen radio. The establishment of the International Radio-medical Center in Rome in 1937 is recognized as the start of what is now called Telematics. NASA was the first organization in the world that made use of it for American astronauts by presenting them remote medical consultations services from the earth. It helps eliminate distance barriers and can improve access to medical services that would often not be consistently available in distant rural communities. It is also used to save lives in critical care and emergency situations. This product of 20^th^ century telecommunication and information technologies, permits communications between patient and medical staff with both convenience and fidelity, as well as the trans mission of medical, imaging and health informatics data from one site to another. Early forms of telemedicine achieved with telephone and radio, advanced diagnostic methods supported by distributed client/server applications, and additionally with tele-medical devices to support in-home care.^1^ Telemedicine can be broken into three main categories: store-and- forward, remote monitoring and (real-time) interactive services. Store-and-forward tele- medicine involves acquiring medical data (like medical images, bio signals etc.) and then transmitting this data to a doctor or medical specialist at a convenient time for assessment offline. It does not require the presence of both parties at the same time. Dermatology, radiology, and pathology are common specialties that are conducive to asynchronous telemedicine. A key difference between traditional inperson patient meetings and telemedicine encounters is the omission of an actual physical examination and history. The 'store-and- forward' process requires the clinician to rely on history report and audio/video information in lieu of a physical examination. Remote monitoring, also known as self-monitoring or testing, enables medical professionals to monitor a patient remotely using various technological devices. This method is primarily used for managing chronic diseases or specific conditions, such as heart disease, diabetes mellitus, or asthma. These services can provide comparable health outcomes to traditional in- person patient encounters, supply greater satisfaction to patients, and may be cost-effective. Interactive telemedicine services provide real- time interactions between patient and provider, to include phone conversations, online communication and home visits. Many activities such as history review, physical examination, psychiatric evaluations and ophthalmology assessments can be conducted comparably to those done in traditional face-to-face visits. In addition, "clinician-interactive" telemedicine services may be less costly than in-person clinical visit.^2^ Telemedicine can be extremely beneficial for people living in isolated communities and remote regions . Patients who live in such areas can be seen by a doctor or specialist, who can provide an accurate and complete examination, while the patient may not have to travel or wait the normal distances or times like those from conventional hospital or GP visits. Recent developments in mobile collaboration technology with the use of hand- held mobile devices allow healthcare professionals in multiple locations the ability to view, discuss and assess patient issues as if they were in the same room.^3^ Telemedicine can be used as a teaching tool, by which experienced medical staff can observe, show and instruct medical staff in another location, more effective or faster examination techniques. It improved access to healthcare for patients in remote locations. "Telemedicine has been shown to reduce the cost of healthcare and increase efficiency through better management of chronic diseases, shared health professional staffing, reduced travel times, and fewer or shorter hospital stays." Several studies have documented increased patient satisfaction of telemedicine over the past fifteen years.^4^ Tele monitoring is a medical practice that involves remotely monitoring patients who are not at the same location as the health care provider. In general, a patient will have a number of monitoring devices at home, and the results of these devices will be transmitted via telephone to the health care provider. Tele-monitoring is a convenient way for patients to avoid travel and to perform some of the more basic work of healthcare for themselves. In addition to objective technological monitoring, most tele monitoring programs include subjective questioning regarding the patient's health and comfort. This questioning can take place automatically over the phone, or tele-monitoring software can help keep the patient in touch with the health care provider. The provider can then make de- cisions about the patient's treatment based on a combination of subjective and objective information similar to what would be revealed during an on-site appointment. Some of the more common things that tele-monitoring devices keep track of include blood pressure, heart rate, weight, blood glucose, and hemoglobin. Tele-monitoring is capable of providing information about any vital signs, as long as the patient has the necessary monitoring equipment at his or her location. Depending on the severity of the patient's condition, the provider may check these statistics on a daily or weekly basis to determine the best course of treatment. Monitoring a patient at home using known devices like blood pressure monitors and transferring the information to a caregiver is a fast growing emerging service. These remote monitoring solutions have a focus on current high morbidity chronic diseases.^5^ Research results showed that telemedicine represented an effective medium for conducting speech assessment in patients with cleft/palate, allowing for increased access to care for undeserved populations.^6^.Studies results represented that e-consultation renders similar outcomes in a selected group of plastic surgery patients. Remote evaluation may assist triage decisions, thereby decreasing emergency room throughput time and office-visit frequency, supplementing satellite facility consultation by plastic surgeons, and providing real time postoperative assessments thereby improving quality and reducing costs.^7^.Some studies have shown that besides telemedicine benefits lie facilitation of disclosure, increased access to services and usefulness concern about privacy and security frame work exist.^8^ Some researches on telemanipulation in urology count such disadvantages like tactile feedback and investment and running costs for telemedicine.^9^ A more recent study in India showed that the development of bio-telemetry will improve healthcare for the rural and remote population and ease the effects of the shortage of rural healthcare professionals.^10^ A study done on critically ill patients, results showed that telemedicine was associated with lower ICU and hospital mortality and felt their quality of life had significantly improved.^11^ Studies done on using telemedicine in high risks pregnancies in Iran, showed that use of telemedicine applications caused increase in recovery rates, decrease in cost.^12^ Overcoming obstacles like, weak tele-communication foundations, medical standards, cultures and cures differences, lack of suitable culture of internet usage, language and literacy differences, lack of an organization for managing telemedicine will pave the way for telemedicine development and execution in Iran.^13^ In a research on “Telemedicine for diabetes support in family doctor’s practices done in Poland, results showed that most of the patients found the system reliable, easy to use and friendly.^14^ In a study done on cultural aspects on tele psychiatry, it was highlighted that cultural background of patients (i.e. their cultural identity) influences their comfort with technology; and the effect of cultural differences on the patient–provider relationship. Cultural differences between patient and provider are often highlighted in telepsychiatry by the patient and provider location (e.g. rural versus urban differences). Familiarity with the rural community and regular contact and feedback are important.^15^ In a more recent study on the effect of ICU telemedicine on mortality and length of stay (2012), implementation of telemedicine and electronic records in the surgical ICU was associated with a profound reduction in severity-adjusted ICU length of stay, ICU mortality, and hospital mortality. However, it is not possible to conclude definitively that the observed associations seen in the SICU were due to the intervention.^16^ Results of a study done (2012) on randomized controlled trial of telemonitoring in older adults with multiple health issues to prevent hospitalizations and emergency department visits, showed that, among older patients, telemonitoring did not result in fewer hospitalizations or ED visits. Moreover; outcomes demonstrated no significant differences between the telemonitoring group and the usual care group. The cause of greater mortality in the telemonitoring group is unknown.^17^

## MATERIALS AND METHODS

Sample selection method was a simple random sampling one. Two hundreds questionnaires were distributed to different hospitals of Tehran Medical Sciences University, Iran. Data collection was carried out from May to August 2012. From these 200 questionnaires, some were valid for the study. Data collection method was a structured interview, document review, field method and questionnaire. In order to ensure respondents did not have any concerns in answering questions or as to the contents of questionnaire, pre-testing and pilot testing were essential before distributing the questionnaires. Pre-testing was done by consulting with some specialists physicians and the university professors. Some modifications and adjustments were made to the original questionnaire. For a better understanding, a pilot study was conducted among 32 specialist physicians. It assured us that the questionnaire was appropriate and the questions were generally understandable. The Cronbach’s alpha value was calculated for reliability of each factor in the questionnaire. Of 32 gathered questionnaires, all values were over the recommended level of 0.7. The final questionnaire consisted of two sections, first section had general questions consist of demographic questions which gathered demographic information such as gender, age, marital status and educational level of respondents. These questions were designed in multiple choice form and respondents can choose which was more applicable for them. The second part of questionnaire that was about telemedicine was designed in 5- points Likert scale. This kind of scaling included strongly disagree, disagree, almost agree, agree and strongly agree. Respondents could choose just one of the option from “1” strongly disagree to “5” strongly agree. Reliability and validity were evaluated for reducing the possibility of getting incorrect answers in the research study. Validity and reliability in this research were done two times. The first time was done by the expert judgment for measuring the quality of the questionnaire, before the distribution. The second time was done after data collection. For that reason, Cronbach test was used for reliability and factor analysis for validity. Reliability analysis allowed studying the properties of measurement scales and the items that make them increase. The reliability was tested two times by Cronbach’s alpha test. At first, it was done on 32 questionnaires as a pilot test and mentioned in pilot test subsection. However, after collecting all data, the reliability of whole data was measured. So the Cronbach’s alpha for each factor in the questionnaire was calculated. The reliability for all factors were more than recommended level of 0.7. Validity was concerned whether the findings were really what they appeared to be. The first time, it was performed on 32 questionnaires which were mentioned on pilot test sub-section. Measurement instrument validity was analyzed by factor analysis. In this study, each measurement criterion was considered as a distinct construct. The most common decision- making technique to obtain factors was to consider factors with Eigen value of over one as significant. According to factor analysis, percentage of total variance were over the recommended level of 50%. In this project, SPSS software (Version 12.0, Chicago, IL, USA) was used for analyzing the collected data. Descriptive statistics like average, variance, standard deviation, frequency distribution, and inferential *statistics *like Chi- Square tests were applied for analyzing the collecting data.

## RESULTS

According to Table 1, 90 (68.2%) of respondents were male and 42 (31.8%) were female. According to Table 2, 4 (3%) of respondents were single and 128 (97%) were married. According to Table 3, 112 (84.4%) of respondents had speciality, 14 (10.6%) fellowships and 6 (4.5%) had professor degree. According to Table 4, 3 (2.3%) of respondents were in 20-30, 59 (44.7%) were 31-40 and 66 (50%) were 41-50 and 4 (3%) were more than 50 years old. According to the results of tables 5 to 10, security affairs and privacy concerns have been known as telemedicine challenges, while being profitable in remote rural regions and in family doctor project, shorter hospital stays, reduced warm ischemic time, decrease in morbidity and mortality rates were some outcomes of applying telemedicine. Culture, language distinctions and level of literacy have been recognized as inhibitors of deploying telemedicine. In regard with treatment/rehabilitation costs, it has been founded cost effective alternative about getting tangible feedback, there has not been any difference between telemedicine and in-person medicine occasions.

**Table 1:** Genre frequency distribution of respondents

**Choice**	**No.**	**%**
Male	90	68.2
Female	42	31.8
Total	132	100

**Table 2:** Marital status frequency distribution of respondents

**Choices**	**No.**	**%**
Single	4	3
Married	128	97
Total	132	100

**Table 3:** Specialty status and frequency distribution of respondents

**Choice**	**No.**	**%**	**Cummulative percen- tage**
Specialist	112	84.8	84.8
Fellowship	14	10.6	95.5
Professor	6	4.5	100
Total	132	100	

**Table 4:** Age frequency distribution of respondents

**Age (years)**	**No.**	**%**	**Cumulative percentage**
20-30	3	2.3	2.3
31-40	59	44.7	47
41-50	66	50	97
>50	4	3	100
Total	132	100	

**Table 5:** Analysis of correlation between using telemedicine frequency and its security considerations

**Using telemedicine frequency security considerations**	**Low**		**Medium**		**Much**		**Very much**		**Total**	
	**No.**	**%**	**No.**	**%**	**No.**	**%**	**No.**	**%**	**No.**	**%**
Disagree	1	6.7	4	26.7	5	33.3	5	33.3	15	100
Almost agree	5	12.8	12	30.8	12	30.8	10	25.6	39	100
Agree	7	11.9	23	39	18	30.5	11	18.6	59	100
Strongly agree	14	73.7	0	0	5	26.3	0	0	19	100
Total	27	20.5	39	29.5	40	30.3	26	19.7	132	100
Significant: 0.001			Df:9				χ^2^: 44.45			

**Table 6:** Analysis of correlation between using telemedicine frequency and shorter hospital stays- reduced warm ischemic time, morbidity and mortality

**Using telemedicine frequency** ** shorter hospital stays-reduced warm ischemic time, morbidity and mortality**	**Low**		**Medium**		**Much**		**Very much**		**Total**	
	**N**	**%**	**N**	**%**	**N**	**%**	**N**	**%**	**N**	**%**
Strongly Disagree	12	46.2	7	26.9	5	19.2	2	7.7	26	100
Disagree	11	15.5	22	31	25	35.2	13	18.3	71	100
Almost agree	4	19	7	33.3	3	14.3	7	33.3	21	100
Agree	0	0	3	21.4	7	50	4	28.6	14	100
Total	27	20.5	39	29.5	40	30.3	26	19.7	132	100
Significant: 0.008			Df: 9				χ^2^: 22.36			

**Table 7:** Analysis of correlation between using telemedicine frequency and culture- language distinctions and level of literacy considerations

**Using telemedicine frequency** ** culture- language distinctions and level of literacy considerations**	**Low**		**Medium**		**Much**		**Very much**		**Total**	
	**No.**	**%**	**No.**	**%**	**No.**	**%**	**No.**	**%**	**No.**	**%**
Strongly disagree	6	33.3	8	44.4	3	16.7	1	5.6	18	100
Disagree	14	36.8	11	28.9	8	21.1	5	13.2	38	100
Almost agree	7	14.9	10	21.3	18	38.8	12	25.5	47	100
Agree	0	0	10	34.5	11	37.9	8	27.6	29	100
Total	27	20.5	39	29.5	40	30.3	26	19.7	132	100
Significant: 0.004			Df:9				χ^2^: 23.8			

**Table 8:** Analysis of using telemedicine and decrease in tactile feedback

**Using telemedicine fre** **quency decrease in tactile feedback**	**Low**		**Medium**		**Much**		**Very much**		**Total**	
	**No.**	**%**	**No.**	**%**	**No.**	**%**	**No.**	**%**	**No.**	**%**
Strongly disagree	3	27.3	2	18.2	2	18.2	4	36.4	11	100
Disagree	15	23.8	22	34.9	17	27	9	14.3	63	100
Almost agree	7	16.3	13	30.2	13	30.2	10	23.3	43	100
Agree	2	13.3	2	13.3	8	53.3	3	20	15	100
Total	27	20.5	39	29.5	40	30.3	26	19.7	132	100
Significant: 0.357			Df: 9				χ^2^: 9.93			

**Table 9:** Analysis of using telemedicine and developing healthcare for rural distant regions and in new “Family physician” project.

**Using telemedicine developing healthcare** **for rural distant regions and in new “Family physician” project**	**Low**		**Medium**		**Much**		**Very much**		**Total**	
	**No.**	**%**	**No.**	**%**	**No.**	**%**	**No.**	**%**	**No.**	**%**
Disagree	8	30.8	7	26.9	8	30.8	3	11.5	26	100
Almost agree	15	33.3	9	20	15	33.3	6	13.3	45	100
Agree	2	5.6	17	47.2	7	19.4	10	27.8	36	100
Almost agree	2	8	6	24	10	40	7	28	25	100
Total	27	20.5	39	29.5	40	30.3	26	19.7	132	100
Significant: 0.007			Df: 9				χ^2^: 22.5			

**Table 10:** Analysis of using telemedicine and increase in treatment/rehabilitation costs

**Using telemedicine** ** increase in treatment/rehabilitation costs**	**Low**		**Medium**		**Much**		**Very much**		**Total**	
	**No.**	**%**	**No.**	**%**	**No.**	**%**	**No.**	**%**	**No.**	**%**
Disagree	7	28	5	20	7	28	6	24	25	100
Almost agree	5	13.9	11	30.6	14	38.9	6	16.7	36	100
Agree	8	22.9	11	31.4	11	31.4	5	17.3	35	100
Strongly Agree	7	19.4	12	33.3	8	22.2	9	25	36	100
Total	27	20.5	39	29.5	40	30.3	26	19.7	132	100
Significant:0/768			Df: 9				χ^2^: 5.72			

## DISCUSSION

The current study counts some benefits of telemedicine, as well as its drawbacks. The first benefit of telemedicine is the leveling of regional differences when same information can be accessed from the next room and from a medical facility several thousand kilometers away. In this way, the quality of medical care would be largely improved because accessibility to special medical care can be extended to rural areas. Telecommunication technologies, such as those used in telemedicine initiatives, have shown to be effective tools for connecting remote sites by opening up new channels for communication, telemedicine connects rural and remote sites with healthcare professionals all parts of the country, overcoming geographical barriers. This can lead to increased communication between health service facilities, and facilitate cross-site and inter-country collaboration and networking. Such collaborations can support healthcare projects like” Family Doctor” recently proposed by the Ministry of Health in Iran. This results are in consistent with previous studies.^1^^,^^3^^,^^4^^,^^6^^,^^10^^,^^14^ According to calculations, the telemedical system is very economically advantageous. Telemedicine could dramatically reduce the overall costs of health services because of its potential to allow a fundamental restructuring of the way health care is delivered. This would principally result from redistributing resources from the hospital environment into primary care. Providing more services in primary care and ultimately in patients’ homes could be considered to be the ultimate goal for health-care delivery and in part this could be facilitated by telemedicine. These results are in line with findings that telemedicine saves the patients money when compared with traditional approaches to providing care.^2^^,^^4^^,^^7^^,^^12^ The third benefit of telemedicine is improved efficiency of medical care by making hospital stays shorter, reducing morbidity and mortality rates. For example, the number of times patients had to be transported was halved. It is a large burden for sick patients to physically go to a hospital. Ideally, a patient could receive a doctor's visit in the comfort of his own home.^2^^,^^4^^,^^7^^,^^10^^,^^11^^,^^12^^,^^16^ Security considerations are a major obstacle to telemedicine uptake. These include an absence of an international legal framework to allow health professionals to deliver services in different parts of a country; a lack of policies that govern patient privacy and confidentiality vis- à- vis data transfer, storage, and sharing between health professionals and jurisdictions; health professional authentication, in particular in e- mail applications; and the risk of medical liability for the health professionals offering telemedicine services. This result is in line in previous researches.^8^ The other challenge of telemedicine revealed some articles is the lack of tactile feedback in comparison to face–to– face medicine. Telemedicine has huge potential to alter medical practice but some improvements are required respect to tactile feedback, instrumentation, telecommunication speed and availability which require clarification. But it is has not been the case for current study. This point is not in line with previous findings.^9^ The last challenge is a complex of human and cultural factors. Some patients and health care-workers resist adopting service models that differ from traditional approaches or indigenous practices, while others lack ICT literacy to use telemedicine approaches effectively. Most challenging of all are linguistic and cultural differences between patients (particularly those underserved) and service, oversight of incompatible cultural subsystems that prevent the transfer of knowledge from one cultural context to another. Without a good understanding of the local context, it may be difficult to integrate telemedicine in a useful way. This result supports previous findings in this regard.^13^^,^^15^ Infrastructure in developing countries like Iran is largely insufficient to utilize the most current internet technologies. By enhancing the information communication technology infrastructure and developing better communication facilities, telemedicine can also add to the better management of scarce medical resources and day-to-day activities. In order to overcome these challenges telemedicine must be regulated by definitive and comprehensive guidelines, which are applied widely, ideally worldwide. Concurrently, legislation governing confidentiality, privacy, access, and liability needs to be instituted. As public and private sectors engage in closer collaboration and become increasingly interdependent in e- Health applications, care must be taken to ensure that telemedicine will be deployed intelligently to maximize health services and optimal quality. It is recommended to deal with telemedicine on the eyes of patients and health officials in future studies.

## Footenote

Please cite this paper as:

Salehahmadi Z, Hajialiasghari F Telemedicine in Iran: Chances and Challenges. World J Plast Surg 2013;2(1):18-25
